# The p53/MDM2/MDMX-targeted therapies—a clinical synopsis

**DOI:** 10.1038/s41419-020-2445-9

**Published:** 2020-04-17

**Authors:** Liren Jiang, Joanna Zawacka-Pankau

**Affiliations:** 1 Department of Pathology Center, Shanghai General Hospital, Shanghai Jiao Tong University School of Medicine, No. 100 Haining Road, Hongkou District, Shanghai, 200080 China; 2 Department of Oncology-Pathology, Karolinska Institute, Stockholm, SE-171 64 Sweden; 3 Faculty of Chemistry, University of Warsaw, 02-093 Warsaw, Poland

**Keywords:** Predictive markers, Translational research

The cost of cancer care accelerates because of the growing number of cancer patients and the increasing expenses of drug innovations. The approval of costly second-line therapies like immune checkpoint inhibitors or CAR-T therapies contributes to this trend. Hence, drug repurposing, which uses drugs originally approved for other indications, is emerging as a necessary approach in cancer care, due to its comparatively low innovation and treatment costs and predictable side-effects^1^.

In this News and Commentary article, we describe our recently published, novel drug repurposing approach for dual targeting of the p53/MDM2 and p53/MDMX interactions followed by the discussion of the selected MDM2/MDMX inhibitors tested in the clinical setting. Lastly, we touch upon recent reports on pharmacological targeting of mutant p53 for improved cancer therapy.

Mutations in the *TP*53 gene occur in more than 50% of all human cancers and lead to p53 loss or mal-adaptivity. Some of the *TP*53 mutations are high penetrance mutations driving tumor development in the hereditary cancer predisposition syndrome called Li-Fraumeni syndrome.

Tumors retaining wild-type (wt) *TP*53 gene, express p53 but the protein is inactivated. MDM2 and MDMX are the most critical regulators of p53 activity^2^.

**Fig. 1:**
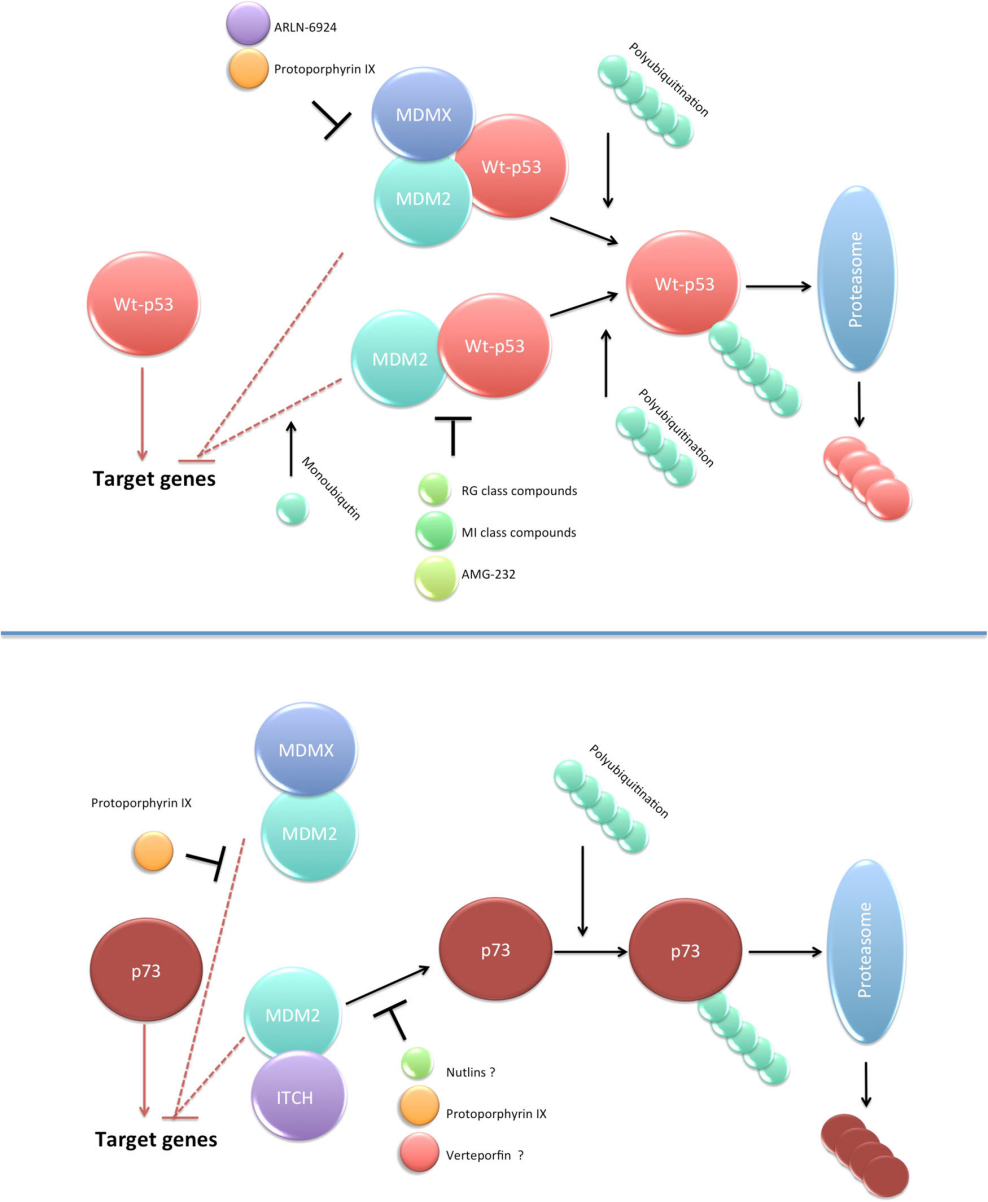
Targeting p53/MDM2/MDMX axis for improved cancer therapy. MDM2 and MDMX are critical negative regulators of p53 and p73 transcription activity and protein stability. Both MDM2 and MDMX bind to p53 N-terminus and inhibit its transcription activity (upper panel). Besides, MDM2 monoubiqutinates p53, which induces p53 nuclear export and the inability to regulate the expression of the target genes. Hetero-dimers of MDMX and MDM2 induce enhanced polyubiquitination of p53 and direct it for proteasomal degradation. Small-molecule MDM2 agonists like RG compounds, MI derivatives or AMG-232 are currently in clinical development. The compounds stabilize p53 and restore the wt-p53 function. Exo-PpIX is the only small molecule inhibiting both p53/MDM2 and p53/MDMX interactions. The only dual inhibitor in clinical trials is a stapled peptide ARLN-6924 (upper panel). Both MDM2 and MDMX also inhibit the transcription activity of p73 tumor suppressor by associating with its N-terminus and inhibiting transcription activity (lower panel). The binding of MDM2 enables the binding of ITCH, E3 ubiquitin ligase of p73 and its proteasomal degradation. In addition to p53, exo-PpIX activates p73 through inhibition of the p73/MDM2 and p73/MDMX. Verteporfin, an analog of PpIX, approved by the FDA to treat age-related macular degeneration in combination with light is a promising candidate for drug repurposing in oncology (http://www.redo-project.org/db/). It was shown to activate p73 in *TP*53 mutant cancer cells presumably through targeting p73/MDM2 interactions similarly to Nutlin^17^. It remains to be elucidated whether Verteporfin is a dual inhibitor of p53/MDM2/MDMX or p73/MDM2/MDMX interactions. A question mark represents not-fully depicted mechanism. Dashed line indicates that the mechanism is not-fully pictured in the figure due to space restrictions.

The MDM2 protein is the p53 E3 ubiquitin ligase that under cellular stress conditions mono- or polyubiquitinates p53. This facilitates p53 nuclear export and inhibition of transcription activity (monoubiquitination) or ubiquitin-dependent proteasomal degradation of p53 (polyubiquitination). The heterodimerization of MDM2 with its homologue, MDMX protein, enhances p53 ubiquitination and degradation. Unlike MDM2, MDMX does not degrade p53, but through the binding to the p53 N-terminus ablates its transcription function^3^ (Fig. 1). The amplification of *MDM*2 and *MDM*4 genes or aberrant expression of their regulators e.g. Wip1, Akt, ATM provoke p53 inhibition in cancer.

One way to restore the p53 pathway in wt-p53 tumors is to block the p53/MDM2 interactions utilizing small molecules and more recently, stapled peptides.

Small molecule protoporphyrin IX (PpIX) is a metabolite of aminolevulinic acid (ALA), a natural heme precursor, which was approved in 1999 as ALA photodynamic therapy (ALA-PDT) to treat actinic keratosis in combination with light. After the ALA administration, PpIX accumulates in the diseased tissue. In ALA-PDT, PpIX is next excited with the light of a wavelength matching the absorption maximum of the compound (*λ* = 410 nm) with 10 J/cm^2^ light dose per treatment. PpIX excitation leads to the accumulation of reactive oxygen species (ROS) and cell death^4^. Interestingly, the outcome of ALA-PDT can be improved by the pretreatment with 5-fluorouracyl (5-FU) because it induces wt-p53, thus predisposes to stress-induced apoptosis. 5-FU also downregulates ferrochelatase, therefore, inhibits the incorporation of Fe^2+^ into PpIX ring and induces massive accumulation of PpIX^5^.

We demonstrated that repurposed, exogenous PpIX (exo-PpIX) induces rapid cancer cell death without light activation^6^. The mechanism is by binding to the p53 N-terminal domain and inhibition of the p53/MDM2 interactions, however, the exact binding site has to be elucidated^7^.

In our latest publication in *Cell Death Discovery*, we showed that exo-PpIX is a dual inhibitor of p53/MDM2 and p53/MDMX interactions and induces apoptosis in B-cell chronic lymphocytic leukemia cells. Using several assays, including yeast-based reporter assay, fluorescent two-hybrid assay (F2H^®^) and immunoprecipitation, we confirmed previous findings demonstrating that PpIX inhibits p53/MDM2 interactions. Besides, exo-PpIX furthers the disruption of p53/MDMX interactions^8^ (Fig. 1).

Comparing to a small molecule RITA, originally reported to display antitumor activity by inhibiting the p53/MDM2 interactions^9^, exo-PpIX inhibits proliferation and induces apoptosis in B-cell chronic lymphocytic leukemia cells (B-CLL) more efficiently and without affecting healthy blood cells^8^. A recent study by Szade et al.^10^ reports that exogenous cobalt PpIX increases the concentration of granulocyte colony stimulating factor (G-CSF) and mobilizes a higher number of mature granulocytes and functional hematopoietic stem cells compared with recombinant G-CSF. Thus, both of the above reported PpIX activities, namely inhibition of p53/MDM2/MDMX interactions and efficient mobilization of cells from the bone marrow to the blood, make exo-PpIX a very promising drug for repurposing in oncology. In particular, it might be of therapeutic benefit to introduce exo-PpIX to treat wt-p53 hematological malignancies. This is due to the high toxicity of current treatments in these cancers reflected by severe neutropenia and increased need for transfusions, which can be decreased with PpIX. Besides, dual targeting of cancers’ vulnerabilities may help to decrease the incidence of the relapsed disease.

Other strategies to target p53/MDM2 interactions converge solely on targeted inhibition of MDM2 protein. The earliest MDM2 antagonist, nutlin (Hoffman‐La Roche), rationally-designed cis‐imidazoline compound, binds to the hydrophobic cleft of the MDM2 and mimicks p53 residues in the MDM2 binding motif; Phe^19^, Trp^23^, and Leu^26^^11^. Idasanutlin (RG73388), the most advanced derivative of nutlin, has shown a promising result in Phase I trial in patients with acute myeloid leukemia (AML)^12^ and has advanced to Phase III trial for relapsed/refractory AML (https://clinicaltrials.gov/show/NCT02545283). AMG-232, a selective and high-affinity piperidinone MDM2 inhibitor, is in Phase I clinical trials for glioblastoma, sarcoma and myeloid malignancies^13^.

Despite the successful advancement to the clinical trial phase, the development of resistance to MDM2 inhibitors, which induces mutations in *TP*53^14^ and triggers an urgent need for the establishment of more effective drug combinations for wt-*TP*53 cancers.

One way to overcome the risk of development of resistance is dual targeting of MDM2 and MDMX, which we study. Several inhibitors of MDMX were reported to date, however, the dual, small-molecule inhibitors of MDM2 and MDMX have not been developed yet. Nowadays, stapled peptides, which bind both to MDM2 and MDMX, see the renascence in targeting wt-p53 tumors. The most advanced, ALRN-6924 peptide, α-helical p53 stapled peptidomimetic, is a potent MDM2/MDMX-antagonist tested in Phase I clinical trials for advanced solid tumors and hematological malignancies^15^.

Despite the efforts, tumors overexpressing MDM2 and MDMX are still a great unmet medical need. Therefore, they constitute high-priority indications for drug repurposing approach described by us^8^.

p53 acts together with p73 and p63 proteins in tumor suppression^4^. In addition to p53, exo-PpIX activates p73 by binding to N-terminal domain and inhibits the p73/MDM2 and p73/MDMX interactions^16^. In our recent work in *Cell Death Discovery*, we confirmed that both p53 and p73 were activated by exo-PpIX in B-CLL cells. In addition to wt-p53, the exo-PpIX activates p73 and induces ROS through inhibition of thioredoxin reductase in mutant *TP*53 cancers^17^. Thus, we reason that exo-PpIX (and Verteporfin) might be a promising drug candidate to target *TP*53 mutant cancers.

Mutant p53-targeting strategies are in clinical development. Small-molecule APR-246 (PRIMA-1^Met^, Aprea Therapeutics, Inc.), a mutant p53 re-activating compound, is the only mutant p53-targeting molecule in the Phase III clinical trial. APR-246 is converted to reactive Michael acceptor, methylene quinuclidinone (MQ). MQ binds covalently to cysteines in p53 via alkylation of thiol groups, stabilizes it and refolds mutant p53 to the wt-like conformation. This restores wt-p53 function and induces cancer cells death^18^. Besides p53, MQ reacts with and inhibits thioredoxin reductase, which induces ROS in cancer cells^19^. It was tested in *TP*53-mutated myelodysplastic syndromes in combination with azacitidine (https://clinicaltrials.gov/show/NCT03745716). Preliminary results of the Phase Ib/II trials in *TP*53-mutated myelodysplastic syndrome in combination with azacitidine showed 74% overall response rate (ORR) and 59% complete remission (CR) rate in 27 evaluable MDS patients (https://clinicaltrials.gov/show/NCT03588078). In a second trial, 88% ORR and 61% CR were reported in 33 evaluable MDS patients. Seventeen (52%) evaluable MDS patients discontinued therapy to pursue stem cell transplant (https://clinicaltrials.gov/show/NCT03072043)^20^.

In conclusion, novel repurposing of PpIX for dual targeting of the p53/MDM2 and p53/MDMX interactions might be a promising alternative to target wt-p53 tumors. Further studies using advanced preclinical models are needed to evaluate its therapeutic potential in *TP*53 mutant cancers.
